# Atypical *NF1* Microdeletions: Challenges and Opportunities for Genotype/Phenotype Correlations in Patients with Large *NF1* Deletions

**DOI:** 10.3390/genes12101639

**Published:** 2021-10-19

**Authors:** Hildegard Kehrer-Sawatzki, Ute Wahlländer, David N. Cooper, Victor-Felix Mautner

**Affiliations:** 1 Institute of Human Genetics, University of Ulm, 89081 Ulm, Germany; 2 Kliniken des Bezirks Oberbayern (KBO), Children Clinical Center Munich, 81377 Munich, Germany; ute.wahllaender@web.de; 3 Institute of Medical Genetics, Cardiff University, Heath Park, Cardiff CF14 4XN, UK; CooperDN@cardiff.ac.uk; 4 Department of Neurology, University Hospital Hamburg Eppendorf, 20246 Hamburg, Germany; v.mautner@uke.de

**Keywords:** neurofibromatosis type 1, NF1, *NF1* microdeletions, genotype/phenotype correlations, *SUZ12*, *CRLF3*, genodermatosis

## Abstract

Patients with neurofibromatosis type 1 (NF1) and type 1 *NF1* deletions often exhibit more severe clinical manifestations than patients with intragenic *NF1* gene mutations, including facial dysmorphic features, overgrowth, severe global developmental delay, severe autistic symptoms and considerably reduced cognitive abilities, all of which are detectable from a very young age. Type 1 *NF1* deletions encompass 1.4 Mb and are associated with the loss of 14 protein-coding genes, including *NF1* and *SUZ12*. Atypical *NF1* deletions, which do not encompass all 14 protein-coding genes located within the type 1 *NF1* deletion region, have the potential to contribute to the delineation of the genotype/phenotype relationship in patients with *NF1* microdeletions. Here, we review all atypical *NF1* deletions reported to date as well as the clinical phenotype observed in the patients concerned. We compare these findings with those of a newly identified atypical *NF1* deletion of 698 kb which, in addition to the *NF1* gene, includes five genes located centromeric to *NF1*. The atypical *NF1* deletion in this patient does not include the *SUZ12* gene but does encompass *CRLF3*. Comparative analysis of such atypical *NF1* deletions suggests that *SUZ12* hemizygosity is likely to contribute significantly to the reduced cognitive abilities, severe global developmental delay and facial dysmorphisms observed in patients with type 1 *NF1* deletions.

## 1. Introduction

It has been estimated that 5–11% of all NF1 patients have large deletions which include the *NF1* gene and its flanking regions at 17q11.2 [1,2,3,4]. These so called ‘*NF1* microdeletions’ are frequently associated with severe clinical manifestations, giving rise to the *NF1* microdeletion syndrome (MIM#613675), which occurs with an estimated incidence of 1:60,000 (reviewed by [5]). Four types of large *NF1* deletion (type 1, 2 and 3 and atypical) have been identified, which are distinguishable in terms of their frequency, size and breakpoint location, by the number of genes deleted and by the frequency of somatic mosaicism with normal cells without the deletion. Most frequent are the type 1 *NF1* deletions which encompass 1.4 Mb and include 14 protein-coding genes as well as five microRNA genes [6,7,8]. Approximately 70–80% of all large *NF1* deletions are of type 1, and in most instances, they occur as germline deletions that are present in all cells of the affected patients [9,10]. The vast majority of type 1 *NF1* deletions result from interchromosomal non-allelic homologous recombination (NAHR) during maternal meiosis [11,12]. NAHR causing type 1 *NF1* deletions occurs between the low-copy repeats NF1-REPa and NF1-REPc. Most type 1 deletions exhibit recurrent breakpoints within the NF1-REPs located in two NAHR hotspots [13,14,15,16,17,18].

Much less frequent than type 1 deletions are those of type 2; approximately 10–15% of large *NF1* deletions are type 2 [9]. Type 2 deletions encompass 1.2 Mb and are associated with the loss of 13 protein-coding genes. In the majority of cases, they are mediated by NAHR and their breakpoints are located within *SUZ12* and its pseudogene *SUZ12P* [19,20]. Type 2 *NF1* deletions are frequently of postzygotic origin, mediated by mitotic NAHR, and hence may occur as mosaic deletions alongside normal cells [19,21,22,23]. Type 3 *NF1* deletions are very rare: they occur in only 1–4% of all patients with large *NF1* deletions. Type 3 deletions encompass 1.0 Mb and are caused by NAHR between NF1-REPb and NF1-REPc [24,25,26]. 

In contrast to type 1, 2 and 3 *NF1* deletions, atypical large *NF1* deletions do not exhibit recurrent breakpoints and are quite heterogeneous in terms of their size and the number of genes located within the deleted region [1,4,6,7,20,25,27,28,29,30,31,32,33,34,35,36,37,38,39,40,41,42,43,44,45,46,47,48,49,50]. It has been estimated that 8–10% of all large *NF1* deletions are atypical [9,25]. Atypical *NF1* deletions may occur as germline mutations but can also be of postzygotic origin [43]. Atypical *NF1* deletions are not only heterogeneous in terms of their length but also in terms of the underlying mutational mechanisms including DNA double strand break repair, aberrant replication and retrotransposon-mediated mechanisms [43]. So far, the clinical phenotype associated with *NF1* microdeletions has mostly been studied in patients with type 1 *NF1* deletions. These patients frequently exhibit more severe clinical manifestations of NF1 as well as features that are not frequently seen in patients with intragenic *NF1* mutations. The type 1 *NF1* deletion-associated phenotype includes facial dysmorphic features, severe global developmental delay, significantly lower full-scale IQ scores, higher neurofibroma burden and an increased risk of malignant peripheral nerve sheath tumours (MPNSTs) as compared to patients with intragenic *NF1* mutations [5,51,52,53,54]. It has been postulated that some of the genes located within the type 1 *NF1* microdeletion region and co-deleted with *NF1* exert an influence on the clinical manifestation of the disease in *NF1* deletion patients. Atypical *NF1* deletions, which encompass only a subset of the 14 protein-coding genes located within the type 1 *NF1* microdeletion region, are of particular interest since the phenotypes expressed by affected patients may facilitate genotype/phenotype correlations in patients with different types of *NF1* microdeletion. However, as yet, only a few patients with atypical *NF1* deletions encompassing a subset of the 14 protein-coding genes have been clinically characterised in any detail. Here, we report on a young patient with an atypical *NF1* deletion that encompasses only 9 of the 14 genes present in the type 1 *NF1* microdeletion region. The clinical phenotype of this patient is potentially informative in the context of genotype/phenotype relationships and the putative modifier role of genes located within the *NF1* microdeletion region. Further, we compare the extent of the deletion in this patient with all atypical *NF1* deletions published to date and the clinical findings in patients with atypical *NF1* deletions that encompass only a subset of the 14 protein-coding genes located within the type 1 *NF1* microdeletion interval.

## 2. Patient and Methods

### 2.1. Genetic Analysis

Male patient 310221 was genetically investigated at the age of 4 years because he exhibited multiple café-au-lait spots, axillary freckling and developmental delay. An atypical *NF1* deletion was detected in patient 310221 by means of CytoScan™ HD array analysis (Affymetrix, Santa Clara, CA, USA). Genomic DNA isolated from the blood of this patient was digested, ligated, amplified, fragmented, labelled and hybridised on the array according the manufacturer’s instructions. The raw data were analysed by means of the Chromosome Analysis Suite software (ChAS, V.3.1.0.15) (Affymetrix, Santa Clara, CA, USA). The deletion was confirmed by multiplex ligation-dependent probe amplification (MLPA) analysis using the SALSA MLPA Probemix P122 (version D2) NF1-area and Probemix P081-D1 NF1 mix 1 as well as P082-C2 NF1 mix 2, (MRC-Holland, Amsterdam, the Netherlands). For MLPA analysis, 100 ng denatured genomic DNA isolated from the blood of the patient was used. MLPA was performed according to the instructions provided by MRC-Holland. MLPA fragments were separated using an ABI Prism 3100 Genetic Analyzer (Applied Biosystems, Foster City, CA, USA). MLPA data analysis was performed with the Coffalyser.Net™ v.1 software (MRC-Holland, Amsterdam, the Netherlands). A reduction of ~0.5 in the peak area values was considered indicative of a deletion of the genomic region represented by the corresponding MLPA probes.

Written informed consent was obtained from the parents of the patient as his legal guardians/representatives. The study was approved by the respective institutional review boards in Hamburg and München and performed in accordance with the Helsinki Declaration and its later amendments.

### 2.2. Investigation of the Cognitive Abilities of Patient 310221

From the age of 3.5 years onwards until the age of 7, the male patient was clinically investigated every 6–9 months at the Children Clinical Center of the Kliniken des Bezirks Oberbayern (KBO) in München, Germany, involving multidimensional field diagnostics in social paediatrics as well as a special consultation for patients with NF1.

The tests performed to investigate the patient’s cognitive abilities and development were the Münchener Funktionelle Entwicklungsdiagnostik for the second and third years of life (MFED) [55,56], the Wechsler Preschool and Primary Scale of Intelligence (WPPSI-III and WPPSI-IV) [57], the Kaufman Assessment Battery for Children (KABC-II) [58], the Reynell Developmental Language Scales (RDLS) [59], the language development tests SETK2 and SETK3–5 [60,61], the Active Vocabulary Test (AWST-R) for children aged 3–5 years [62] and the Beery–Buktenica Developmental Test of Visual–Motor Integration (Beery VMI) [63].

## 3. Results

### 3.1. Clinical Investigation of Patient 310221

The male patient was born spontaneously at term as the second child of healthy, unrelated parents. At birth, his weight was 3520 g (P 56), his length was 49 cm (P 11) and his head circumference was 35 cm (P 42). Results of newborn screening as well as hearing tests were normal. Muscular hypotonia or feeding problems were absent during the neonatal period. The patient was able to sit on his own by the age of 6–7 months, started to crawl at the age of 10 months and to walk at the age of 14 months in a timely manner. However, developmental delay affecting primarily speech and coordination was suspected when the patient was approximately 2 years old. He spoke his first specific words at the age of 18–24 months and sentences comprising two words at the age of 2 years. His progress in acquiring vocabulary was slow. The patient had multiple café-au-lait spots over 5 mm in diameter, disseminated mainly on his trunk and not restricted to one body segment. Additionally, axillary freckling was noted on both sides and hence the patient fulfilled the NIH diagnostic criteria for NF1. Nevus anemicus was observed on his chest and upper arms. From the age of 3.5 years onwards until the age of 7 years, the boy was clinically investigated every 6–9 months. Neither neurofibromas nor xanthogranulomas were detected at the age of 3.5 years. He had mild pectus excavatum and pes planovalgus of both feet. Generalised muscular hypotonia was diagnosed at the age of 3.5 years, which persisted in later years. The patient was investigated by cranial MRI at the age of 3.5 and 7 years, which did not disclose intracranial tumours, inflammation, structural brain abnormalities, macrolesions or foci of abnormal signal intensity (FASI). Neither thickening of the optic nerves nor optic gliomas were detected by cranial MRI. The subarachnoid spaces were of normal size. Audiological evaluation of the patient indicated normal hearing. 

At the age of 3.5 and 4.6 years, the patient was investigated by an ophthalmologist using optical coherence tomography (OCT) and slit lamp examination. Neither Lisch nodules nor choroidal anomalies were detected. The patient had macrocephaly, and from the age of 4.6 years onwards, small body size was noted, which persisted in later years (Supplementary Table S1).

At the age of 5.7 years, spinal MRI was performed. Neither intraspinal nor paravertebral tumours were detected. Mild scoliosis was noted as well as a kyphosis of the pelvic spine. Osseous lesions such as sphenoid wing dysplasia and anterolateral bowing of the tibia were not observed. 

Abdominal ultrasound investigation performed at the age of 6.1 years did not indicate internal tumours. However, at this age, a small cutaneous neurofibroma was detected on his chest. The patient had mild hypertelorism, thinning of the eyebrows at the inner roots and a slightly low hairline. However, he did not exhibit facial dysmorphic features frequently seen in patients with type 1 *NF1* deletions, including coarse facial appearance, downward-slanting palpebral fissures, long philtrum, micrognathia and broad nasal bridge. At the time of writing this report, the patient was 7 years old.

### 3.2. Development and Cognitive Abilities

Delays in the development of language as well as gross motor skills were first suspected when the patient attended a day nursery at the age of 2 years. To assess this more thoroughly, he was examined by developmental tests performed at the age of 3.5 years (MFED and WPPSI-III). The subtests assessing his performance skills revealed an IQ of 93, indicating average nonverbal cognitive abilities for his age. However, in subtests assessing his passive vocabulary, he showed below average performance. The patient was delayed in his development of expressive and receptive language by one year according to RDLS for children at the age of 1–6 years. The SETK2 language development test, performed when the patient was 3.5 years old, indicated a delay in vocabulary acquisition of 0.5–1 years. The patient also exhibited deficits in grammar, articulation and oral motor function. Attention deficits and inadequate emotional control in situations outside of his immediate family environment were also noted. To support his development, the patient started to attend an integrative kindergarten for disabled and nondisabled children and received comprehensive ergotherapy, special education as well as speech therapy once per week. 

In response to the therapeutic intervention, the patient made good progress over the following year. The language development tests SETK3–5 and AWST-R, performed when the patient was 4.2 years old, indicated considerable improvements in receptive language development and vocabulary acquisition. His capabilities in these areas were estimated to be equal those of children aged 3.6–4 years, indicating that his developmental delay in these skills had been reduced. He was raised to be bilingual, with German being the main language and English his second language. Nevertheless, at the age of 4.2 years, he still showed deficits in expressive language skills, including grammar and articulation. The orofacial hypotonia persisted.

At the age of 4.6 years, his mental processing and cognitive abilities were evaluated by means of KABC-II (Supplementary Table S2). The Fluid-Crystallised Index (FCI), the general intelligence composite score of the patient, was 87, which is at the lower end of the range of normal FCI values (85–115) determined for healthy children at the age of 3–6 years. The adjusted mean FCI in 505 children at the age of 3–6 years was 101.6 [58]. The patient’s Mental Processing Index (MPI), the global intelligence score based on the Luria model of the KABC-II, was 91, which is within the normal range. The adjusted mean MPI in 505 healthy children was 101 [58]. The evaluation of the boy’s drawing capabilities revealed a developmental delay of approximately one year. Taken together, the tests performed when the patient was 4.2–4.6 years old indicated normal cognitive abilities. His skills in receptive language had improved considerably, but he still showed mild developmental delays in expressive language, fine-motor skills and visuospatial functioning. Attention deficit problems and hyperactivity persisted, which impaired his performance. 

The patient continued to attend the integrative kindergarten and received special education and speech therapy on a continual basis. At the age of 5.5 years, his cognitive development was assessed by means of WPPSI-IV. The full-scale IQ of the patient was 99, which is clearly within the normal range. Considerable variation was, however, observed in the results of the subtests (Supplementary Table S3). The language development tests SETK3–5 and AWST-R indicated receptive and expressive language skills in the normal range according to his age. In the subtest of the KABC-II, which assesses auditory short-term memory including number recall, the patient performed well for his age. Hence, the patient had caught up in his development of expressive and receptive language. Indeed, the progress made by the patient was rated as very positive. All in all, his intellectual and language abilities were stable at an average level. However, mild deficits were still observed in fine motor and graphomotor skills as determined by the Beery VMI test. Although his overall assessment at the age of 5.5 years was very positive, distractibility and attention deficits persisted. Furthermore, deficits in controlling impulses and social integration were noted.

At the age of 6.1 years, the patient was investigated again by means of the KABC-II developmental test. The values obtained on the different scales varied considerably (Supplementary Table S2). The FCI of the patient was 88, which is at the lower boundary of the range of normal FCI values (85–115). Deficits in attention and executive functions impaired his performance. The patient performed well in the kindergarten; his socioemotional competence had improved, but he showed deficits in certain aspects of social contact behaviour with other children. He was successful in preschool tasks, was able to write several words and spoke in English and German. 

At the age of 6.7 years, the patient started to attend primary school in a class that included the provision of special needs education. He performed well, was successfully integrated into the class and was eager to learn. However, he was easily distractible and showed self-centred and sometimes socially restrained behaviour, which raised the suspicion of the development of an autism spectrum disorder.

### 3.3. Characterisation of the Deletion in Patient 310221

**Figure 1:**
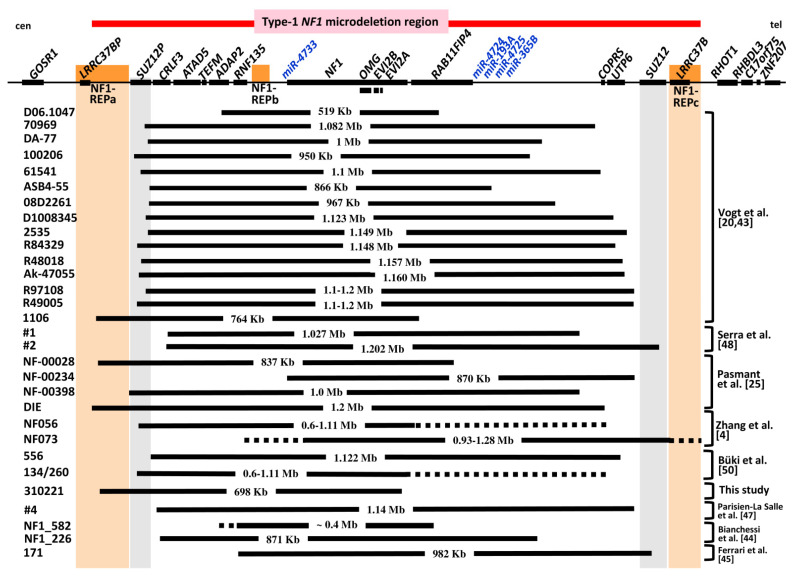
Schema of the type 1 *NF1* microdeletion region, which includes 14 protein-coding genes as well as the *SUZ12P* pseudogene and 5 microRNA genes. The relative locations of these genes are indicated by black rectangles. The low-copy repeats NF1-REPa and NF1-REPc (shaded) are located at the boundaries of the type 1 *NF1* microdeletion region. Indicated also is the extent of the known atypical *NF1* deletions of group #2, which are represented by black horizontal bars. The atypical *NF1* deletions of group #2, which exhibit breakpoints located within the boundaries of the type 1 *NF1* microdeletion region, are smaller than the much more frequent type 1 *NF1* deletions and do not encompass all of the 14 protein-coding genes located within the type 1 *NF1* microdeletion region. A total of 30 group #2 atypical *NF1* deletions have been reported to date, inclusive of patient 310221 described in this study (patient acronyms are given on the left). The extent of these deletions is indicated by horizontal bars. Details of the clinical phenotypes associated with the atypical *NF1* deletions included in the studies of Pasmant et al. [25], Vogt et al. [20,43] and Bianchessi et al. [44] were not reported.

An atypical *NF1* deletion encompassing the *NF1* gene and its flanking regions was identified by microarray analysis, performed when the patient was 4 years old. According to this analysis, the deletion extends over position 28,997,893–29,695,563 (Human Genome Build GRCh37/hg19) and encompasses 698 kb (ISCN 2020: arr[GRCh37] 17q11.2(28997893-29695563)x1). The centromeric deletion breakpoint is located within NF1-REPa, and the telomeric deletion breakpoint within intron 57 of the *NF1* gene, as confirmed by MLPA analysis. The deletion identified in patient 310221 encompasses the *NF1* gene, its three embedded genes and the five genes located centromeric to *NF1*, namely *CRLF3, ATAD5, TEFM, ADAP2* and *RNF135* (Figure 1). Three of these nine genes (namely *NF1*, *OMG* and *ATAD5*) are loss-of-function intolerant, as determined by the metric ‘probability of being loss-of-function intolerant (pLI)’ (Table 1). The pLI score partitions genes into loss-of-function intolerant (pLI ≥ 0.9) or tolerant (pLI ≤ 0.1) [64]. For *ATAD5* and *NF1*, the pLI score is 1.00, indicating extreme intolerance to loss-of-function variants. 

Neither array analysis nor MLPA analysis of blood and saliva-derived genomic DNA of the patient indicated the presence of somatic mosaicism with normal cells. The deletion of patient 310221 was not identified in the blood of his parents or his healthy sister and is hence considered to have occurred de novo.

### 3.4. Comparison of the Deletion in Patient 310221 with Previously Reported Atypical NF1 Deletions

So far, 61 atypical *NF1* deletions have been reported [1,4,6,7,20,25,27,28,29,30,31,32,33,34,35,36,37,38,39,40,41,42,43,44,45,46,47,48,49,50]. These 61 atypical *NF1* deletions can be classified into two groups: The first encompasses very large deletions extending beyond one or both of the boundaries of the 1.4 Mb type 1 *NF1* microdeletion region which is flanked by NF1-REPa and NF1-REPc. In total, 31 such large atypical *NF1* deletions have been identified, which together constitute group #1 (Table 2). 

The second group of atypical *NF1* deletions, termed group #2 deletions, exhibit breakpoints that are located within the type 1 *NF1* microdeletion region. The deletion of patient 310221 analysed in this study belongs to this group #2 atypical *NF1* deletions, which are smaller than 1.4 Mb in size and not associated with the loss of all 14 protein-coding genes located within the type 1 *NF1* microdeletion interval. Thus, group #2 atypical deletions encompass only a subset of these 14 genes. Taken together, 30 group #2 atypical *NF1* deletions have been reported to date, including patient 310221 (Figure 1, Table 3). Remarkably, 15 (50%) of these 30 group #2 atypical *NF1* deletions exhibit centromeric breakpoints located within the *SUZ12P* pseudogene. A total of 5 of the 30 group #2 atypical *NF1* deletions have centromeric breakpoints located within NF1-REPa, including the deletion of patient 310221 reported here. Thus, *SUZ12P* and NF1-REPa represent genomic regions predisposed to chromosomal breakage causing atypical *NF1* deletions. 

**Table 1:** Protein-coding genes located within the 1.4 Mb type 1 *NF1* microdeletion region. The genes rendered hemizygous in patient 310221 as a consequence of the 698 kb atypical deletion are marked in bold type.

Official HGNC Gene Symbol	MIM#	Official Gene Name	pLI Score
***CRLF3***	614853	cytokine receptor like factor 3	0
***ATAD5***	609534	ATPase family, AAA domain containing 5	1.00
***TEFM***	616422	transcription elongation factor, mitochondrial	0.51
***ADAP2***	608635	ArfGAP with dual PH domains 2	0.00
***RNF135***	611358	ring finger protein 135	0.00
***NF1***	162200	neurofibromin	1.00
***OMG***	164345	oligodendrocyte myelin glycoprotein	0.97
***EVI2B***	158381	ecotropic viral integration site 2B	0.06
***EVI2A***	158380	ecotropic viral integration site 2A	0.00
*RAB11FIP4*	611999	RAB11 family interacting protein 4	0.99
*COPRS*	616477	coordinator of PRMT5 and differentiation stimulator	0.25
*UTP6*	-	UTP6 small subunit processome component	0
*SUZ12*	613675	SUZ12 polycomb repressive complex 2 subunit	1.00
*LRRC37B*	616558	leucine rich repeat containing 37B	0.01

According to GnomAD v2.1.1/GnomAD SVs v2.1. The gnomAD browser (https://gnomad.broadinstitute.org/ accessed on 15 October 2021) provides the constraint metric termed ‘probability of loss-of-function’ (pLI). To determine the pLI metric, the observed and expected variant counts for a given gene are considered. The closer the pLI value is to 1, the more loss-of-function intolerant the gene appears to be. A pLI score ≥ 0.9 is indicative of genes that are predicted to be intolerant of loss-of-function variants [64].

**Table 2:** Large atypical *NF1* deletions with one or both breakpoints located beyond the boundaries of the type 1 *NF1* microdeletion region as defined by flanking NF1-REPa and NF1-REPc (group #1 atypical NF1 deletions).

Patient ID	Deletion Size in Mb	References
UWA 106-3	3.2–3.7	[6,27,28,31]
UWA 155-1	2.1–2.7	[6,31]
ID806	~7	[29,37]
3724A	2.0–3.1	[1,31]
UWA 113-1	~1	[6]
BUD	4.7	[7,31,50]
BL	3	[30]
6	3	[32]
118	1–2	[33]
442	2	[35,50]
806	5.5	[25,36]
T165	>2.2	[36,37]
282775	>1.33	[36,37]
T145	1.61–1.75	[36,37]
SNF1-2	~1.3	[38]
SNF1-3	1.84–2.8	[38]
552	2.7	[39]
DUB	7.6	[40]
NF00358	1.2	[41]
D05.2678	5.9	[43]
D0801587	2	[43]
619	3	[43,51]
ID not specified	2.8	[42]
NF040	1.27–1.46	[5]
NF076	1.26–1.63	[5]
NF1_31	1.8	[44]
NF1_505	1.1	[44]
NF1_724	>1.6	[44]
ID not specified	1.695	[46]
2019	1.26–1.63	[49]
125	1.6	[50]

**Table 3:** Features of the 30 atypical deletions of group #2. Indicated are the locations of the deletion breakpoints, the methods used to analyse the deletions and the number of functional *SUZ12* gene copies in the respective patients. nd: not determined; BS-PCR: breakpoint-spanning PCR; MLPA: multiplex ligation-dependent probe amplification.

Patient ID	Centromeric Breakpoint	Telomeric Breakpoint	Method of Analysis	*SUZ12* Copies	Deletion Size	Mosaic	Reference
D06.1047	*ADAP2*	*RAB11FIP4*	BS-PCR, MLPA	2	519,291 bp	yes	[43]
70969	*SUZ12P*	*RAB11FIP4* and *COPRS*	BS-PCR, MLPA	2	1,082,491 bp	no	[43]
DA-77	*SUZ12P*	between *RAB11FIP4* and *COPRS*	BS-PCR, MLPA	2	1,001,546 bp	yes	[43]
100206	*SUZ12P*	between *RAB11FIP4* and *COPRS*	BS-PCR, MLPA	2	950,940 bp	yes	[43]
61541	*SUZ12P*	between *COPRS* and *UTP6*	BS-PCR, MLPA	2	1,105,242 bp	yes	[43]
ASB4-55	*SUZ12P*	between *RAB11FIP4* and *COPRS*	BS-PCR, MLPA	2	866,769 bp	yes	[43]
08D2261	*SUZ12P*	between *RAB11FIP4* and *COPRS*	BS-PCR, MLPA	2	976,455 bp	yes	[43]
D1008345	*SUZ12P*	*UTP6*	BS-PCR, MLPA	2	1,123,78 bp	no	[43]
2535	*SUZ12P*	between *UTP6* and *SUZ12*	BS-PCR, MLPA	2	1149,077 bp	no	[43]
R84329	*SUZ12P*	*UTP6*	BS-PCR, MLPA	2	1,148,828 bp	yes	[43]
R48018	*SUZ12P*	between *UTP6* and *SUZ12*	BS-PCR, MLPA	2	1,157,378 bp	no	[43]
Ak-47055	*SUZ12P*	between *UTP6* and *SUZ12*	BS-PCR, MLPA	2	1,160,989 bp	yes	[43]
R97108	*SUZ12P*	between *UTP6* and *SUZ12*	MLPA	2	1.1–1.2 Mb	nd	[20]
R49005	*SUZ12P*	between *UTP6* and *SUZ12*	MLPA	2	1.1–1.2 Mb	nd	[20]
1106	NF1REPa	*RAB11FIP4*	BS-PCR, MLPA	2	764,080 bp	no	[43]
#1	*CRLF3*	28 kb centromeric to *COPRS*	microarray	2	1,027,355 bp	no	[48]
#2	*CRLF3*	*SUZ12*	microarray	1	1,202,659 bp	no	[48]
NF00028	*LRRC37BP*	*RAB11FIP4*	microarray	2	837 Kb	nd	[25]
NF00234	*RNF135*	between *UTP6* and *SUZ12*	microarray	2	870 Kb	nd	[25]
NF00398	*LRRC37BP*	between *RAB11FIP4* and *COPRS*	microarray	2	1.0 Mb	nd	[25]
DIE	NF1-REPa	between *COPRS* and *UTP6*	microarray	2	1.2 Mb	nd	[25]
NF056	*SUZ12P*	between *NF1* exon 57 and *UTP6*	MLPA	2	0.6–1.11 Mb	nd	[4]
NF073	between *RNF135* and *NF1* exon 1	between *LRRC37B* and *ZNF207*	MLPA	1	0.93–1.28 Mb	nd	[4]
556	between *SUZ12P* and *CRLF3*	*UTP6*	microarray, MLPA	2	1,122,447 bp	yes	[50]
134/260	*SUZ12P*	between *NF1* exon 58 and *UTP6*	MLPA	2	0.6–1.11 Mb	no	[50]
310221	NF1-REPa	intron 57 of *NF1*	microarray, MLPA	2	698 Kb	no	this study
#4	*CRLF3*	between *UTP6* and *SUZ12*	microarray	2	1,144,007 bp	nd	[47]
NF_582	between *ADAP2* and *RNF135*	between *NF1* exons 57 and 58	MLPA	2	~0.4 Mb	nd	[44]
NF_226	*CRLF3*	between *RAB11FIP4* and *COPRS*	microarray	2	871 Kb	nd	[44]
171	*RNF135*	*SUZ12*	microarray	1	981,763 bp	nd	[45]

Only 2 of the 30 known group #2 atypical *NF1* deletions may be associated with the loss of the same nine genes as observed in patient 310221, namely the deletions in patients 134/260 and NF056 (Figure 1). However, the deletions in these patients were analysed exclusively by MLPA, and the telomeric deletion breakpoints cannot be assigned with any accuracy because of the large gap between the MLPA probes located at the end of the *NF1* gene and the *UTP6* gene. It is therefore unclear whether the deletions in these patients also encompass the *RAB11FIP4* and *COPRS* genes (Figure 1). Patients 134 and 260 possess the same 0.6–1.11 Mb deletion. Patient 134 is a 40-year-old woman who passed on the deletion to her 8-year-old son, patient 260. However, patient 134 is also likely to have inherited the deletion since her mother was affected by NF1 [50]. The deletion identified in patients 134 and 260 differs from the 698 kb deletion of patient 310221 in terms of the location of the centromeric breakpoint. Two further group #2 atypical *NF1* deletions, those in patients 1 and 556, are of interest in terms of comparing their extent with the deletion in patient 310221, since clinical data from the respective patients have been published. Patient 1, reported by Serra et al. [48], harbours an atypical *NF1* deletion of 1.027 Mb encompassing 10 genes (Figure 1). The deletion in patient 1 leads to hemizygosity of the same nine genes as observed in patient 310221. However, in addition to these nine genes, the *RAB11FIP4* gene is also deleted in patient 1. Patient 556, reported by Büki et al. [50], harbours an atypical *NF1* deletion of 1.12 Mb associated with the loss of 13 genes. The *SUZ12* gene is not included in the deletion interval in this patient. If all 30 atypical group #2 deletions are considered, only three of them are associated with the loss of one copy of *SUZ12* (Table 3).

### 3.5. Genotype/Phenotype Correlations in Patients with Atypical NF1 Deletions of Group #2

Group #2 atypical *NF1* deletions can inform genotype/phenotype correlations if they are associated with only some of the clinical features frequently observed in patients with the larger type 1 *NF1* deletions. However, details of the clinical phenotype are only available for 9 of the 30 patients with group #2 atypical *NF1* deletions (Table 4). In six of these nine patients, the deletion does not encompass *SUZ12*, whereas in three patients, *SUZ12* is either present only in one copy due to the deletion or functionally inactivated by the telomeric deletion breakpoint (Table 3 and Table 4). 

Developmental delay was observed in patient 310221 reported here as well as in patient 1 analysed by Serra et al. [48]. Patient 1 was investigated at the age of 4 and 6 years. She attended primary school, and dysgraphia was noticed after several months. Hyperactivity was also evident. She had a full-scale IQ of 88 which is in the normal range, but heterogeneous results were observed in the different subtests. Her performance IQ was 122, which is in the high normal range. However, her verbal IQ was 71, which is well below average. The speech impairment of patient 1 was more severe than the delay in the development of receptive and expressive language of patient 310221. Nevertheless, neither patient showed the global, very severe developmental delay in numerous areas as frequently observed in children with type 1 *NF1* deletions.

In addition, patients 556 and 134 with group #2 deletions did not exhibit severe developmental delay (Table 4). By contrast, patient 260 who had the same deletion as his mother (patient 134), showed severe delay in cognitive development at the age of 8 years [50]. The severity of the developmental delay in patient 260 is, however, difficult to estimate. For this patient, neither IQ values nor detailed information about specific tests performed to assess different areas of developmental delay have been reported. It is also unknown whether patient 260 received any therapy to improve his skills. As shown for the patient 310221 reported here, therapeutic intervention may help to reduce developmental delays quite considerably. 

Mild hypertelorism was noted in patient 310221, and hypertelorism was also observed in patients 134 and 260. However, other facial dysmorphic features frequently seen in patients with type 1 *NF1* deletions, including coarse face, downward-slanting palpebral fissures and broad nasal bridge, were not observed in patient 310221 or in any other patient with an atypical *NF1* deletion of group #2 and two copies of the *SUZ12* gene (Table 4). 

Importantly, severe cognitive impairment associated with an IQ < 70 was not observed in any of those patients with a group #2 atypical *NF1* deletion who had been clinically analysed in greater detail and whose deletion did not encompass *SUZ12*. For three of these patients with atypical group #2 deletions that did not encompass *SUZ12*, IQ values were assessed and found to be in the normal range (Table 4). By contrast, patient 2, reported by Serra et al. [48], had severe global developmental delay and an FSIQ of 55. The telomeric breakpoint of the deletion in patient 2 is located within the *SUZ12* gene. Hence, this deletion is associated with the functional inactivation of *SUZ12*.

**Table 4:** Clinical features observed in patients with group #2 atypical *NF1* deletions.

Clinical Features	Patients								
	556	134	260	310221	#1	#2	NF056	NF073	171
	m, 10y	f, 40y	m, 8y	m, 7y	f, 4y	f, 3y	f, 60y	f, 25y	m, 3y
Broad nasal bridge	nd	nd	nd	−	−	+	nd	nd	nd
Downward slanting palpebral fissures	nd	nd	nd	−	−	nd	nd	nd	nd
Hypertelorism	−	+	+	+, mild	−	+	nd	nd	nd
Facial asymmetry	−	−	−	−	−	nd	nd	nd	nd
Coarse face	−	−	−	−	−	nd	nd	nd	nd
Micrognathia	nd	nd	nd	−	−	nd	nd	nd	nd
Broad neck	−	−	−	−	−	+	nd	nd	nd
Large hand and feet	−	−	−	−	−	nd	nd	nd	nd
Excess soft tissue on hands	−	−	−	−	−	nd	nd	nd	nd
Café-au-lait spots	+	+	+	+	+	+	+	+	+
Freckling	+	+	+	+	+	+	+	+	+
Lisch nodules	−	−	−	−	+	nd	nd	nd	−
Tall stature/overgrowth	−	−	−	−	−	−	nd	nd	+
Subcutaneous neurofibromas	−	+	−	−	−	−	nd	nd	nd
Cutaneous neurofibromas	−	−	−	−	−	−	+	+	nd
Externally visible plexiform neurofibromas	−	−	−	−	−	−	nd	nd	nd
Spinal neurofibromas	−	nd	nd	−	nd	nd	nd	nd	nd
Delay in development	−	−	+	+	+	global, +	nd	nd	+
Learning difficulties	−	−	−	+	+	+	nd	nd	nd
Speech difficulties	−	−	−	+	+	nd	nd	nd	nd
IQ	89	nd	nd	93	88	55	nd	nd	nd
Attention deficit and hyperactivity	−	−	−	+	+	nd	nd	nd	nd
Autism spectrum disorder	nd	nd	nd	+	nd	nd	nd	nd	nd
Scoliosis	−	+	−	+, mild	+, severe	nd	−	−	nd
Pectus excavatum	−	−	+	+	−	nd	−	−	nd
Bone cysts	−	−	−	−	−	nd	−	−	nd
Other bone abnormalities	−	−	−	pes planus	bilateral calcaneovalgus	genu valgum, pes planus	−	−	nd
Joint hyperflexibility	−	−	−	nd	nd	nd	nd	nd	nd
Macrocephaly	−	−	+	+	nd	+	nd	nd	−
Muscular hypotonia	−	−	−	+	+	−	nd	nd	+
MPNSTs	−	−	−	−	−	−	−	−	nd
T2 hyperintensities	−	nd	+	−	−	+	nd	nd	+
Optic gliomas	+	−	+	−	−	−	nd	nd	nd
***SUZ12*** **copy number**	**2**	**2**	**2**	**2**	**2**	**1**	**2**	**1**	**1**

nd: not determined; −: absent; +: present; y: years; m: male; f: female. MPNST: malignant peripheral nerve sheath tumour. Patients 556, 134 and 260 were reported by Büki et al. [50], patients 1 and 2 by Serra et al. [48], patients NF056 and NF073 by Zhang et al. [4], patient 171 by Ferrari et al. [45]. The telomeric breakpoints of the deletions in patient 2 and 171 are located within the *SUZ12* gene. Hence, these deletions were associated with the functional inactivation of *SUZ12*. Patient 2 exhibited severe global developmental delay and dysmorphic features, including broad forehead, dysplastic and low-set ears with thick helix, synophrys, receding orbital roof with exophthalmus, malar hypoplasia and long and prominent philtrum.

## 4. Discussion

In contrast to type 1 *NF1* deletions, atypical *NF1* deletions are much less frequent. In total, 61 atypical *NF1* deletions have been reported to date, including the patient described here (Table 2, Figure 1). Atypical *NF1* deletions are either larger or smaller than type 1 *NF1* deletions. In total, 31 large atypical *NF1* deletions have been reported which extend beyond one or both boundaries of the type 1 *NF1* microdeletion region (group #1; Table 2). The deletion of additional genes located beyond the boundaries of the type 1 *NF1* microdeletion region is expected to give rise to additional clinical symptoms or an even more complex clinical phenotype than generally observed in patients with type 1 *NF1* deletions. More informative with regard to genotype/phenotype correlations are those atypical *NF1* deletions, which are smaller than 1.4 Mb, each encompassing only a subset of the 14 protein-coding genes located in the type 1 *NF1* microdeletion region. As yet, 30 such atypical *NF1* deletions have been identified; these are termed group #2 atypical deletions (Figure 1, Table 3 and Table 4). Unfortunately, clinical data are only available for nine of the patients with these deletions, and only five of them have been characterized in any detail (Table 4).

The atypical deletion of patient 310221 reported here is remarkable since it spans only 698 kb and encompasses nine genes. Patient 310221 exhibited multiple café-au-lait spots and freckling as well as developmental delay first noted when he was 2 years old. At the age of 3.5 years, he showed developmental delay in expressive as well as receptive language and, to a minor degree, in motor skills. He was considered to be delayed in his development by approximately one year. However, therapeutic intervention helped him to catch up, and his deficits diminished considerably over the following years. The developmental delay observed in patient 310221 was much less severe than the global developmental delay frequently observed in children with type 1 or very large atypical *NF1* microdeletions (reviewed in [5]). Severe global developmental delay in language and motor skills has been observed in 28 (93%) of 30 children with *NF1* microdeletions [51]. These delays were already apparent at a young age (1–3 years) and persisted during later childhood. A total of 2 of the 30 children had very large atypical *NF1* deletions, extending beyond the boundaries of the *NF1* microdeletion region, whereas 28 children had type 1 deletions [51]. These findings imply that severe global developmental delay is frequent in children with type 1 *NF1* microdeletions. By contrast, the developmental delay observed in patient 310221 reported here was restricted to specific areas and therapeutic intervention helped to reduce the delay so that he could catch up with his peers. 

It should be appreciated that developmental delays are not rare among children in the general NF1 population [65,66,67,68,69]. Delays in at least one of eight areas, including fine motor, gross motor, receptive language, expressive language, math/premath ability, reading/pre-reading, self-help and socioemotional development, were observed in 68% of children with NF1 unselected for *NF1* mutation type [65]. Significant developmental abnormalities were found in the areas of fine motor (35%), gross motor (52%) and math/premath ability (31%) [65]. At least 54% of children with NF1 older than 4 years exhibited lower scores for motor proficiency indicating poorer motor development [70]. In a study of 39 toddlers (21–30 months of age), psychomotor scores were found to be significantly lower for those with NF1 compared to those without NF1 [71]. Hence, developmental delays often present early in development in children with NF1. 

Wessel et al. [66] investigated the progression of deficits caused by developmental delays in children with NF1 over time. Analysis of the total delays in several areas revealed that the number of areas delayed increased with age. The mean proportion of areas delayed was 22% in infants, 28% in preschool and 47% in school-age children with NF1 [66]. Closely related to motor performance is visuospatial functioning, which is frequently impaired in children with NF1 (reviewed by [72]). Numerous studies have indicated cognitive impairments and learning disabilities in 30–65% of all children with NF1 [72]. Children with NF1 frequently exhibit impairment in motor control and learning accompanied by substantial problems in visuomotor integration [73,74]. Nevertheless, the developmental delays observed in children with NF1 and intragenic *NF1* mutations are much less severe than the global developmental delays affecting multiple areas seen in many children with large *NF1* microdeletions [51]. The developmental delay in patient 310221 reported here appears to be within the range of developmental delays frequently seen in children with NF1 and intragenic *NF1* mutations. Although patient 310221 showed a delay in the development of expressive and receptive language, he responded well to therapeutic intervention and was able to improve his deficits considerably, acquiring language skills within the normal range for his age. He also improved in terms of his gross motor skills, but mild deficits in fine motor skills have persisted. 

Not much is known about the areas of developmental delay and their progression over time in other patients with atypical *NF1* deletions of group #2, which encompass only a subset of genes within the type 1 *NF1* microdeletion region. Only for seven patients with group #2 atypical *NF1* deletions has information about the presence of developmental delay been published (Table 4). Remarkably, female patient 1 with a 1.027 Mb atypical *NF1* deletion reported by Serra et al. [48], also exhibited developmental delay mainly associated with speech impairment. Her FSIQ was in the normal range. The cognitive abilities of patient 310221 reported here were also in the normal range as measured by several tests (Supplementary Table S3). The full-scale IQ (FSIQ) of patient 310221 at the age of 5.5 years was 99 whereas the mean FSIQ in 470 children with intragenic *NF1* mutations was 87.7 [52]. Previous studies have indicated that the FSIQ in children from the general NF1 population is in the normal range, although somewhat lower than in children of comparison groups (usually around 90, instead of the normative mean of 100) [72,75]. By contrast, patients with type 1 *NF1* deletions have significantly lower FSIQ scores than individuals carrying intragenic pathogenic *NF1* variants or the general NF1 population [51,52,76,77]. The mean FSIQ of patients with type 1 *NF1* deletions analysed in these studies ranged from 71.2–77.9 (Supplementary Table S4). 

The normal cognitive abilities of patient 310221 and his comparatively moderate developmental delay are suggestive of a possible genotype/phenotype correlation. Thus, genes located telomeric to *NF1,* and not encompassed by the 698 kb atypical *NF1* deletion of patient 310221, may contribute to the significantly reduced FSIQ and severe global developmental delays frequently seen in patients with type 1 *NF1* deletions. The deletion in patient 310221 does not encompass the five genes *RAB11FIP4, COPRS*, *UTP6, SUZ12* and *LRRC37B.* These genes are located telomeric to *NF1* and are included in the type 1 *NF1* microdeletion region (Figure 1). Remarkably, only two of these five genes, *RAB11FIP4* and *SUZ12*, are loss-of-function intolerant, implying that their haploinsufficiency in patients with *NF1* microdeletions is highly likely to have severe pathological consequences. *RAB11FIP4* expression is highest in the brain, particularly in the cortex and frontal cortex [78]. However, it is still unknown if and how *RAB11FIP4* hemizygosity contributes to the phenotype associated with large *NF1* microdeletions. 

In contrast to *RAB11FIP4*, much more information is available for *SUZ12* which renders *SUZ12* an important putative modifier of the type 1 *NF1* microdeletion-associated phenotype. *SUZ12* has a pLI score of 1.00, indicative of extreme intolerance to loss-of-function variants (Table 1) [64]. Hemizygosity of *SUZ12* has been shown to be associated with an increased risk of MPNSTs in patients with type 1 *NF1* microdeletions [79,80,81]. Importantly, patients with pathogenic variants located within *SUZ12* exhibit pre- and postnatal overgrowth, facial dysmorphic features, musculoskeletal abnormalities and developmental delay/intellectual disability [82,83]. SUZ12 is one of the core components of the polycomb repressive complex 2 (PRC2), an epigenetic regulator with H3K27 methyltransferase activity. PRC2 is involved in gene silencing and the control of many different processes during cellular differentiation (reviewed by [84]). Mutations in the genes encoding other components of PRC2, namely *EZH2* and *EED*, also lead to overgrowth, macrocephaly, advanced bone age, variable intellectual disability and distinctive facial features [84]. Since intellectual disability is quite common in patients with mutations of genes encoding PRC2 components, it is likely that the loss of *SUZ12* contributes to the cognitive disabilities and severe developmental delay seen in patients with type 1 *NF1* deletions hemizygous for *SUZ12*. The normal cognitive abilities of patient 310221 and other patients with atypical group #2 deletions who are not hemizygous for *SUZ12* are in accordance with this putative genotype/phenotype correlation (Table 4). 

Remarkably, patients with intragenic *SUZ12* mutations exhibit an overgrowth phenotype [82,83,84]. Childhood overgrowth is also frequent in patients with large *NF1* deletions. In contrast to the short stature observed in most patients with intragenic *NF1* mutations, tall stature in adults and overgrowth during childhood have been reported in patients with *NF1* microdeletions [5,25,77,85,86,87,88]. The loss of the *RNF135* gene located centromeric to *NF1* within the type 1 *NF1* deletion region has been suggested to be associated with the overgrowth seen in patients with *NF1* microdeletions [89]. However, tall stature and childhood overgrowth have also been reported in patients with *NF1* deletions that do not encompass the *RNF135* gene but are associated with the loss of *SUZ12* [24,25,26,89]. Thus, it may be inferred that the heterozygous loss of *SUZ12* resulting in *SUZ12* haploinsufficiency is likely to contribute to the overgrowth phenotype observed in patients with type 1 *NF1* microdeletions. This conclusion is supported by the observation that *SUZ12* is not included within the deletion interval in patient 310221 who is small for age. Overgrowth or tall stature was also not reported for other patients with atypical *NF1* deletions of group #2 (Table 4). 

Patients with intragenic *SUZ12* mutations exhibit facial dysmorphic features similar to those observed in many patients with type 1 *NF1* deletions including hypertelorism, broad nasal bridge and downward-slanting palpebral fissures [82,83]. Importantly, facial dysmorphic features are rare in patients with intragenic *NF1* mutations. Hemizygosity of *SUZ12* is likely to contribute to the facial dysmorphic features of patients with type 1 *NF1* deletions. This conclusion is supported by the absence of facial dysmorphism, as frequently seen in patients with type 1 *NF1* deletions, in patient 310221 who has two copies of *SUZ12*. However, facial dysmorphism is not apparent in all patients with type 1 *NF1* deletions or at least not to the same extent. It follows that the causes of facial dysmorphism in patients with type 1 *NF1* deletions are likely to be complex, being influenced by *SUZ12* hemizygosity as well as additional factors. 

Many studies have identified impairment of executive functions and working memory in patients with NF1 [90]. An increased prevalence of attention deficit hyperactivity disorder (ADHD) in children with NF1 has been reported [91,92,93,94,95]. ADHD impairs the daily life functioning of many children with NF1 quite considerably [96]. ADHD is also frequent in patients with type 1 *NF1* deletions [77] and has been diagnosed in 15 (88%) of 17 children and adolescents with type 1 *NF1* deletions [51]. Patient 310221, reported here, exhibited attention deficits which impaired his learning over and above the direct impact on his cognitive and adaptive skills. The frequency of attention deficits in patients with atypical *NF1* deletions has not so far been investigated in any detail and further studies are required to evaluate the impact of these deficits on performance and development in this group of patients. 

In addition to ADHD, a high incidence of autism has been observed in patients with NF1 [92,97,98,99,100]. Autism spectrum disorder (ASD), with a phenotypic profile similar to idiopathic autism, is observed in at least 25% of children with NF1, whereas an additional 20% of children with NF1 exhibit partial ASD features [101]. Autism-related symptoms also appear to be very frequent in patients with *NF1* microdeletions. In a previous study, we observed mild to moderate autistic symptomatology in 15 (71%) of 21 children with type 1 *NF1* deletions, which was significantly more frequent than in the general NF1 population [51]. Autistic symptomatology and hyperactivity were also noticed in patient 310221. At the age of 7 years, the onset of an autism spectrum disorder was suspected. 

A specific role in the development of the *NF1* microdeletion-associated phenotype and in particular autism has recently been demonstrated for the cytokine receptor-like factor 3 (*CRLF3*) gene located within the *NF1* microdeletion region. *CRLF3* is deleted in patient 310221 reported here (Figure 1). Induced pluripotent stem cell forebrain cerebral organoids (hCOs), isolated from patients with type 1 *NF1* microdeletions, display both neural stem cell proliferation and elevated neuronal abnormalities such as dendritic maturation deficits. Whilst increased neuronal stem cell proliferation has been shown to result from decreased NF1/RAS regulation, the neuronal differentiation, survival and maturation defects of these hCOs are caused by reduced *CRLF3* expression and impaired RhoA signalling [102]. This role of *CRLF3* was confirmed by the analysis of hCOs, isolated from a patient with an atypical *NF1* deletion which did not include the *CRLF3* gene. These hCOs did not show abnormalities of neuronal survival, differentiation and maturation [102]. Further, these authors identified 7 of 17 NF1 patients with an increased autistic trait burden who harboured a germline putatively pathogenic missense variant within the *CRLF3* gene (c.1166T > C, p.Leu389Pro) present in addition to pathogenic variants in *NF1*. Taken together, these findings suggest an essential role for *CRLF3* in both human brain development and autism [102]. Consequently, the loss of the *CRLF3* gene in the patient described here may have contributed to his autistic symptoms. Further analyses of patients with different types of *NF1* microdeletion will confirm or refute the possible contribution of the *CRLF3* gene to the deletion-associated phenotype.

Atypical *NF1* deletions of group #2, such as the 698 kb deletion observed in patient 310221, are potentially very informative in terms of genotype/phenotype correlations. However, the possibility that atypical *NF1* deletions can be associated with mosaicism with normal cells not harbouring the deletion must be considered. Vogt et al. [43] observed mosaicism with normal cells in 10 (59%) of 17 patients with atypical *NF1* deletions. In 6 of these 10 patients, mosaicism was analysed by FISH which allowed the determination of the proportion of cells harbouring the deletion. In the blood samples of these six patients, the proportion of cells with the deletions was 70%, 75%, 80%, 93%, 96% and 98%, respectively. In three (50%) of the six patients, mosaicism was also detected by MLPA, which is considered to have an intrinsic detection limit of ~10% (reviewed in [10]). In other words, low-grade mosaicism with normal cells present at a proportion lower than ~10% cannot be detected by MLPA. Since FISH on blood cells was not performed in patient 310221, low-grade mosaicism with normal cells cannot be unequivocally excluded even though MLPA and microarray analysis did not give any indication of mosaicism with normal cells. Patient 310221 does not show features frequently seen in patients with type 1 *NF1* deletions, such as global severe developmental delay, cognitive disability, overgrowth and facial dysmorphism, which are all detectable at a very young age. Even though we cannot completely exclude the possibility that normal cells lacking the deletion are present in low proportions and could have influenced the clinical phenotype in patient 310221, his comparatively mild clinical phenotype is likely to be attributable to the presence of two copies of the *SUZ12* gene, which do not reside within the deletion interval in this patient.

## 5. Conclusions

Group #2 atypical *NF1* deletions, encompassing only a subset of the 14 protein-coding genes located within the type 1 *NF1* microdeletion region, have the potential to inform genotype/phenotype correlations and facilitate the identification of modifying genes. Patient 310221 analysed here harbours a group #2 atypical *NF1* deletion which encompasses the *CRLF3* gene but not *SUZ12*. The relatively mild clinical phenotype of this patient suggests that the retention of *SUZ12* may have ameliorated what might otherwise have been a more severe clinical phenotype. Indeed, the relatively mild clinical phenotype of group #2 atypical *NF1* deletion patients with two copies of *SUZ12* suggests that *SUZ12* hemizygosity is highly likely to influence the more severe clinical phenotype observed in patients with type 1 *NF1* microdeletions characterized by facial dysmorphic features, reduced cognitive abilities and severe global developmental delay, all of which were absent in patient 310221. The co-deletion of *CRLF3* may contribute to the clinical manifestations (particularly the autistic symptomatology) in those patients whose *NF1* microdeletions encompass this gene. However, further studies are necessary to investigate this in greater detail. To date, genotype/phenotype correlations for group #2 atypical *NF1* deletions are limited by the scarcity of clinical details available for many of the reported patients and by limited breakpoint definition due to the use of MLPA instead of microarray analysis. Moreover, information about possible mosaicism with cells not harbouring the deletion is not available for many patients with atypical *NF1* deletions. The future analysis of genetically and clinically well-characterized patients with atypical *NF1* deletions will serve to further clarify the role of the loss of genes such as *SUZ12* and *CRLF3* for the *NF1* microdeletion-associated phenotype.

## Data Availability

Data are contained within the article or Supplementary Material.

## Supplementary Materials

The following are available online at https://www.mdpi.com/article/10.3390/genes12101639/s1, Table S1: Physical measurements of patient 310221; Table S2: Patient 310221 was investigated by means of the Kaufman Assessment Battery for Children (KABC-II). The KABC-II measures 5 scales according to the Cattell-Horn-Carroll (CHC) model. The results of the subtests are indicated as index-values of the patient. The summary of all index-values from the scales is represented by the Fluid-Crystallised Index (FCI), the general intelligence composite score of KABC-II according to the CHC model; Table S3: The cognitive abilities of patient 310221 were evaluated by the Kaufman Assessment Battery for Children (KABC-II) and the Wechsler Preschool and Primary Scale of Intelligence. FCI: Fluid-Crystallised Index (FCI), the general intelligence composite score of KABC-II according to the CHC model; Table S4: Full scale IQ (FSIQ) in patients with NF1 microdeletions analysed by Descheemaeker et al., Mautner et al., Ottenhoff et al. and Kehrer-Sawatzki et al. The 17 patients with NF1 microdeletions analysed by Ottenhoff et al. indicated in this table do not include the patients analysed by Descheemaeker et al. Six patients included in the study of Kehrer-Sawatzki et al. had already been analysed previously by Mautner et al.; these six patients are not included in the 17 NF1 microdeletion patients analysed by Mautner et al. listed below.
