# Supplementary material for: Atypical NF1 Microdeletions: Challenges and Opportunities for Genotype/Phenotype Correlations in Patients with Large NF1 Deletions
Source: Genes (Basel). 2021 Oct 19;12(10):1639. doi: 10.3390/genes12101639 (PMC8535936; doi:10.3390/genes12101639)
Supplement: Supplementary file 1 [file genes-12-01639-s001.zip › genes-1399180-supplementary.pdf]

Supplementary Tables:

Supplementary Table S1: Physical measurements of patient 310221

Age in years	Body measures (P percentile)	
	weight in kg	height in cm
3.5	15 (P 39)	99.5 (P 43)
4.6	16.7 (P 28)	104 (P 17)
5.5	17.9 (P 15)	109.4 (P 11)
6.1	18.3 (P 9)	110 (P 4)
7	20 (P 9)	118 (P 14)

Supplementary Table S2: Patient 310221 was investigated by means of the Kaufman Assessment Battery for Children (KABC-II). The KABC-II measures 5 scales according to the Cattell-Horn-Carroll (CHC) model. The results of the subtests are indicated as index-values of the patient. The summary of all index-values from the scales is represented by the Fluid-Crystallised Index (FCI), the general intelligence composite score of KABC-II according to the CHC model.

Scales of the KABC-II	Capabilities assessed by the subtests of the scales	Index-values at the age of 4.6 years[a]	Index-values at the age of 6.1 years[b]	FCI at the age of 4.6 years	FCI at the age of 6.1 years
Learning/Glr	long-term memory and retrieval	100	87		
Sequential/Gsm	short term memory	100	91		
Simultaneous/Gv	visual processing and conceptual thinking	85	87		
Planning/Gf	pattern reasoning	85	n.d.		
Knowledge/Gc	expressive vocabulary and verbal knowledge	n.d.	97		
				87 (95% CI: 82-93)	88 (95% CI: 83-93)

n.d. not determined

a: At the age of 4.6 years, the index-values of the patient were 100 on the scales Learning/Glr and Sequential/Gsm, assessing long-term and short-term memory. These index-values of 100 are within the normal range for children of his age. However, on the scale Simultaneous/Gv, investigating his skills in visual processing and conceptual thinking, as well as on the scale Planning/Gf assessing pattern recognition and reasoning, the patient exhibited deficits and achieved index-values of only 85, which are below average.

b: At the age of 6.1 years, the patient was investigated again by means of the KABC-II developmental test. The values obtained on the different scales varied considerably. He displayed average performance in tests assessing verbal knowledge and short term memory including number recall. However, he showed mild deficits in tests that assessed long term memory due to attention deficits and distractibility.

Supplementary Table S3: The cognitive abilities of patient 310221 were evaluated by the Kaufman Assessment Battery for Children (KABC-II) and the Wechsler Preschool and Primary Scale of Intelligence. FCI: Fluid-Crystallised Index (FCI), the general intelligence composite score of KABC-II according to the CHC model.

Test performed	Age of the patient in years	IQ or FCI
WPPS-III	3.5	Performance IQ 93
KABC-II	4.6	FCI 87 (95% CI: 82-93)
WPPS-IV[a]	5.5	Full scale IQ 99 (95% CI: 93-105)
KABC-II	6.1	FCI 88 (95% CI: 83-93)

a: Considerable variation was observed in the results of the subtests underlying the composite full-scale IQ. The patient performed below average in the subtest that investigated attention regulation and speed of processing. In subtests assessing visuospatial processing and fluid reasoning, the patient exhibited above average index values of 118 and 117, respectively. In the subtest evaluating receptive and expressive language skills, the patient achieved an index value of 93 which is within the normal range.

Supplementary Table S4: Full scale IQ (FSIQ) in patients with *NF1* microdeletions analysed by Descheemaeker et al. [76], Mautner et al. [77], Ottenhoff et al. [52] and Kehrer-Sawatzki et al. [51]. The 17 patients with *NF1* microdeletions analysed by Ottenhoff et al. [52] indicated in this table do not include the patients analysed by Descheemaeker et al. [76]. Six patients included in the study of Kehrer-Sawatzki et al. [51] had already been analysed previously by Mautner et al. [77]; these six patients are not included in the 17 *NF1* microdeletion patients analysed by Mautner et al. [77] listed below.

	**Descheemaeker et al. [76]**	**Mautner et al. [77]**	**Kehrer-Sawatzki et al. [51]**	**Ottenhoff et al. [52]**
Total number of patients analysed	11	17	24	17
Mean FSIQ	76.0	77.9	77.7	71.2
(SD; 95% CI)	(6.9; 71.4–80.6)	(14.3; 71.1–84.7)	(12.8; 72.6–82.8)	(10.3; 66.3–76.1)
FSIQ range	65–85	49–104	51–110	60–92
Number of patients with FSIQ <70	2 (18%)	6 (35%)	5 (21%)	7 (41%)
Number of patients with FSIQ ≥70 – <85	8 (73%)	4 (24%)	12 (50%)	8 (47%)
Number of patients with FSIQ <85	1 (9%)	2 (12%)	1 (4%)	0
Number of patients with FSIQ >85	0	5 (29%)	6 (25%)	2 (12%)

All 11 patients analysed by Descheemaeker et al. [76] and all 17 patients analysed by Mautner et al. [77] had type-1 *NF1* deletions of 1.4-Mb. Among the 17 patients investigated by Ottenhoff et al. [52], nine patients had type-1 *NF1* deletions, two deletions were atypical, one deletion was type-2 and five deletions were not further characterized with regard to their length. Three of the 24 patients analysed by Kehrer-Sawatzki et al. [51] had atypical *NF1* microdeletions of 4.7-Mb, 3-Mb and 2-Mb, respectively.
